# Fall risk assessment dataset: older-adult participants undergoing the time up and go test

**DOI:** 10.1016/j.dib.2023.109653

**Published:** 2023-10-06

**Authors:** Wisanu Jutharee, Chatchai Paengkumhag, Warissara Limpornchitwilai, Wen Tao Mo, Jonathan H. Chan, Tanagorn Jennawasin, Boonserm Kaewkamnerdpong

**Affiliations:** a Institute of Field Robotics and Automation, King Mongkut's University of Technology Thonburi, Bangkok, Thailand; b Biological Engineering Program, Faculty of Engineering, King Mongkut's University of Technology Thonburi, Bangkok, Thailand; c Innovative Cognitive Computing (IC2), School of Information Technology, King Mongkut's University of Technology Thonburi, Bangkok, Thailand; d Engineering Science, Faculty of Applied Science and Engineering, University of Toronto, Toronto, Canada; e Department of Control System and Instrumentation Engineering, Faculty of Engineering, King Mongkut's University of Technology Thonburi, Bangkok, Thailand

**Keywords:** Aging, Signal data, IMU sensors, Walking analysis

## Abstract

This article presents a dataset comprising signal data collected from Inertial Measurement Unit (IMU) sensors during the administration of the Time Up and Go (TUG) test for assessing fall risk in older adults. The dataset is divided into two main sections. The first section contains personal, behavioral, and health-related data from 34 participants. The second section contains signal data from tri-axial acceleration and tri-axial gyroscope sensors embedded in an IMU sensor, which was affixed to the participants' waist area to capture signal data while they walked. The chosen assessment method for fall risk analysis is the TUG test, requiring participants to walk a 3-meter distance back and forth. To prepare the dataset for subsequent analysis, the raw signal data underwent processing to extract only the walking periods during the TUG test. Additionally, a low-pass filter technique was employed to reduce noise interference. This dataset holds the potential for the development of effective models for fall risk detection based on insights garnered from questionnaires administered to specialists who observed the experiments. The dataset also contains anonymized participant information that can be explored to investigate fall risk, along with other health-related conditions or behaviors that could influence the risk of falling. This information is invaluable for devising tailored treatment or rehabilitation plans for individual older adults. The complete dataset is accessible through the Mendeley repository."

Specifications Table

**Table:** 

Subject	Computer Science
Specific subject area	Signal processing, fall risk detection
Data format	The participants’ data are raw data in comma-separate values (CSV) format. The signal data contains raw data and processed data from the IMU sensor in CSV format.
Type of data	The participants’ data contained character and numeric values. Signal data from the IMU sensor while performing the Timed Up and Go (TUG) test was then generated in the form of numeric values.
Data collection	Participant data and IMU sensor signals were collected. Participant information obtained through interviews, questionnaires, and assessments, focused on personal and health details. Signal data from an IMU sensor, linked to an ESP8266 controller within the measuring device, captured raw signals of acceleration and gyroscope sensors during the Timed Up and Go (TUG) test. Signal data covered three TUG rounds. The data was stored on a memory card connected to the measuring device's controller. Raw data underwent pre-processing, eliminating extraneous signals during idle periods before and after the task.
Data source location	Institution: King Mongkut's University of Technology Thonburi City: Bangkok Country: Thailand
Data accessibility	Repository name: Fall Risk Assessment Dataset: Older-Adult Participants undergoing the Time Up and Go Test Data identification number: doi:10.17632/78mw556vjc Direct URL to data: https://data.mendeley.com/datasets/78mw556vjc/

## 1 Value of the Data

• The world as a whole is becoming an aging society; the proportion of the older population is up to 34% of the total population [1]. Older people suffer from declines in both physical performance and mental abilities, leading to high fall risks. Accidents from falling are one of the common causes of death in the older population [2]. Older people with a history of falling tend to have a lower quality of life. Due to the fall, they could develop health problems, both mentally and physically [3]. It is a general consensus that prevention of falls is better than cure [4].
• In order to prevent falls and to reduce the risk of falling, proper assessment tools that are fast at prediction with high accuracy are required; being able to assess risk early will have a positive effect on the prevention of falling for the elderly. Effective fall prevention requires expert knowledge and the acquisition of accurate information to identify potential issues in the monitoring process of the preventive tools [5]. Nowadays, technology such as portable sensors plays a crucial role in both risk assessment, such as early detection, and monitoring [6].
• The dataset consisted of two parts: participants’ information (e.g., gender, age, medical conditions, fall history, and specialists’ assessment results) and Inertial Measurement Unit (IMU) sensor data captured from participants' waist while they were performing the Timed Up and Go (TUG) test, which is a simple test used to measure fall risk for older adults. The IMU sensor measures linear acceleration and rotational rate by using accelerometers and gyroscopes, respectively. The dataset can be employed to investigate fall risk as well as other health conditions or behaviors that could impact the risk of falling.
• The dataset can be used to develop an effective fall risk detection model by combining sensor data and insights from specialists gathered through questionnaires, resulting in a high-performance solution for early detection. Cross-disciplinary studies might combine these data with other datasets (e.g., genetic data and medical records) to explore complex relationships between genetics, health conditions, and mobility. Moreover, clinicians can use insights from datasets to tailor rehabilitation programs for elderly patients.

## 2 Data Description

The collected data is securely stored within the Mendeley Data, which comprises two distinct folders. The first folder, “*Participant_Information*,” encompasses the questionnaire, clinical assessments, and participants' data. The second folder, designated as “*Signal_Data*,” includes the numerical data derived from the Inertial Measurement Unit (IMU) sensors of each participant during the execution of the Timed Up and Go (TUG) test. The raw signal data is also provided in the folder “*Raw_Signal_Data*.”

The questionnaire for collecting participants' data was designed to understand their backgrounds and behaviors. The file is named “*BasicQ.pdf*.” The questions in this file pertain to age, gender, weight, height, education level, exercise behavior, smoking behavior, and underlying diseases (U/D). In the context of mental health, we utilized the Depression Screening Questionnaire, referred to as Q9, found in the file “*Q9.pdf*,” to assess the propensity for depressive conditions. For evaluating basic activities of daily living among the participants, we employed the BADL assessment tool, detailed in the file “*BADL.pdf*.” To assess the risk of cognitive impairment or dementia and to evaluate cognitive abilities, we used the MoCA assessment outlined in the file “*MOCA.pdf*.” Moreover, the “*Obs_checklist.pdf*” was employed by three experts to assess the physical capabilities of participants while performing the TUG (Timed Up and Go) test. All the questionnaires and assessment test results mentioned have already been compiled in the “*ParticipantsData.csv*” file.

The IMU signal data is derived from the acceleration and gyroscope measurements captured while participants perform the Timed Up and Go (TUG) test. The sensor model used in this study is the MPU6050 IMU Module. The signal data is separated into two folders. The “*Raw_Signal_Data*” folder consists of 34 sub-folders, each named after the unique ID of a participant (e.g., “ps01” corresponds to participant number one). Within each participant's ID folder, three CSV files indicate the raw signal from each round of the TUG test as “*Trial 0*,” “*Trial 1*,” and “*Trial 2*.” The processed signal data is organized within the “*Signal_Data*” folder. This folder consists of 34 sub-folders, each named after the unique ID of a participant, the same as the raw signal data folder. Within each participant's ID folder, there are three sub-folders: “*Trial 0*,” “*Trial 1*,” and “*Trial 2*.”x Each of these trial folders contains signal data files captured during different rounds of the TUG test. Specifically, within each trial folder, there are six CSV files:

1. “*All_pxx.csv*”: This file includes signal data from all phases of the TUG test. Here, “xx” represents the participant's ID number ranging from 01 to 34.
2. “*Turn 1.csv*”: This file contains signal data from the turning phase at the turning point of the TUG test.
3. “*Turn2.csv*”: Signal data from the turning phase at the start point (when the participant turns to sit down) is stored in this file.
4. “*Walk 1.csv*”: Signal data from the walking phase, which extends from the start point to the turning point, is recorded in this file.
5. “*Walk 2.csv*”: Signal data from the walking phase, covering the path from the turning point back to the start point, is stored in this file.
6. “*Sit.csv*”: This file comprises signal data from the sitting phase, which encompasses the period when the participant transitions from standing to sitting down. The test concludes after the participant is fully seated.

Each raw signal record file includes attributes outlining the characteristics of the captured signals, as described in Table 1. The attributes of the processed signal data are described in Table 2.

**Table 1:** The description of a data record from the raw signal data.

Column	Description
a_x	Acceleration along the x-axis of the sensor device
a_y	Acceleration along the y-axis of the sensor device
a_z	Acceleration along the z-axis of the sensor device
g_x	Angular velocity around the x-axis of the sensor device
g_y	Angular velocity around the y-axis of the sensor device
g_z	Angular velocity around the z-axis of the sensor device

**Table 2:** The description of a data record from the processed signal data.

Column	Description
Subject	ID of participant (ps01 to ps34)
Trial Num	TUG trial round (0 = first round, 1 = second round, 2 = third round)
Duration	The time taken to perform the TUG test.
a_x	Acceleration along the x-axis of the sensor device
a_y	Acceleration along the y-axis of the sensor device
a_z	Acceleration along the z-axis of the sensor device
g_x	Angular velocity around the x-axis of the sensor device
g_y	Angular velocity around the y-axis of the sensor device
g_z	Angular velocity around the z-axis of the sensor device
acc_tot	The magnitude of linear acceleration
a_x jerk	Derivative of a_x
a_y jerk	Derivative of a_y
a_z jerk	Derivative of a_z
g_x jerk	Derivative of g_x
g_y jerk	Derivative of g_y
g_z jerk	Derivative of g_z
acc_tot jerk	Derivative of the magnitude of linear acceleration

## 3 Experimental Design, Materials and Methods

We invited participants aged between 60 and 70 years to take part in the study, adhering to specific inclusion criteria. These criteria encompassed the following aspects: the capability to independently perform daily life activities (BADL > 12), the absence of depressive symptoms (9Q < 7), proficiency in Thai language communication, and willingness to provide informed consent for participation in the research. The exclusion criteria were the following: refraining from medication affecting the nervous system and abstaining from beverages containing caffeine and/or alcohol for at least 6 h before the experiment; ensuring a minimum of 6 h of restful sleep; absence of visual impairments, color blindness, hearing challenges, speech impairments, and movement restrictions that could impede participation; the ability to provide comprehensive data; and a commitment to not withdrawing from the study.

Regarding determining the appropriate sample size for this study, we performed sample size estimation in the context of two independent groups, as described by the Lemeshow method [7]. This calculation drew upon the variance data derived from the study conducted by Zakaria et al. [8], which investigated fall risk assessment using the Timed Up and Go (TUG) test, specifically focusing on the Mean Step Time per individual step. Upon applying the aforementioned methodology to gage the sample size requirements, it was established that a minimum of 30 participants was necessary for constructing the sample groups effectively. These participants were subsequently divided into two groups, each consisting of 15 individuals, to ensure robust and statistically sound representation for analytical purposes.

In total, our study includes 34 participants. They are divided into 2 equal groups: the group of healthy older adults, the participants who had not experienced any falls in the preceding year, and the group of older adults at risk of falling; this group included older adults who had encountered falls within the past year. The subsequent sections outline the particulars of the experimental process.

## 4 Materials

### 4.1 Time Up and Go (TUG) Protocol

The TUG test stands out as an effective and dependable technique, enabling accurate and precise screening of senior individuals susceptible to falls. Its simplicity facilitates a quick testing process, rendering it applicable for fall assessment across diverse elderly populations—within residences, communities, or healthcare facilities. This inclusiveness extends to elderly persons grappling with neurological, skeletal, and muscular conditions. This evaluation adheres to the guidelines outlined by the CDC (Centers for Disease Control and Prevention), encompassing the measurement of time and recording of gait patterns across a 3-meter distance. Should the senior individual take 12 s or more to complete the test, it raises a flag for potential fall risk. The TUG procedure is as follows:

• Instruct the patient:
  When I say “Go,” I want you to:
  1. Stand up from the chair.
  2. Walk to the line on the floor at your normal pace.
  3. Turn.
  4. Walk back to the chair at your normal pace.
  5. Sit down again.
• On the word “Go,” begin timing.
• Stop timing after the patient sits back down.
• Record duration.

### 4.2 Wearable Device Prototype for Data Collection

In this study, we developed a prototype of the portable device for data collection. This prototype encompasses an array of measurement instruments strategically positioned at the waist. The ensemble comprises the following components:

• Inertial Measurement Unit (IMU) Sensor Module: The MPU6050 IMU sensor module demonstrates the capability to gage acceleration and orientation angles along the x, y, and z axes. It transmits continuous streams of acceleration and orientation signals across time, thereby facilitating further processing by the control system. This functionality is instrumental in identifying falls and conducting comprehensive assessments of fall-related risks.
• Control Module - ESP8266 Model: The ESP8266 control module serves as the hub for signal processing and communication. It receives both the acceleration and the angular velocity signal from the IMU sensor module and then samples the signal into a set of discrete data points. This control module can be used to process the data points in order to construct a model for fall detection as well as to establish benchmarks for evaluating the likelihood of falls. Notably, the module boasts compatibility with wireless communication technologies such as Wi-Fi and Bluetooth, enhancing its versatility. In the current experiment, the continuous-time signals are sampled at a frequency of 20 times per second (20 Hz).
• Data Backup Device - SD Card Module: A pivotal component of the setup is the SD Card Module. It stores the discrete data points, which are sampled from the continuous-time signals obtained from the IMU sensor module. The SD card module can store a data set measured for a minimum duration of 10 min using a sampling frequency of 20 Hz, thereby ensuring comprehensive coverage.

**Fig. 1:**
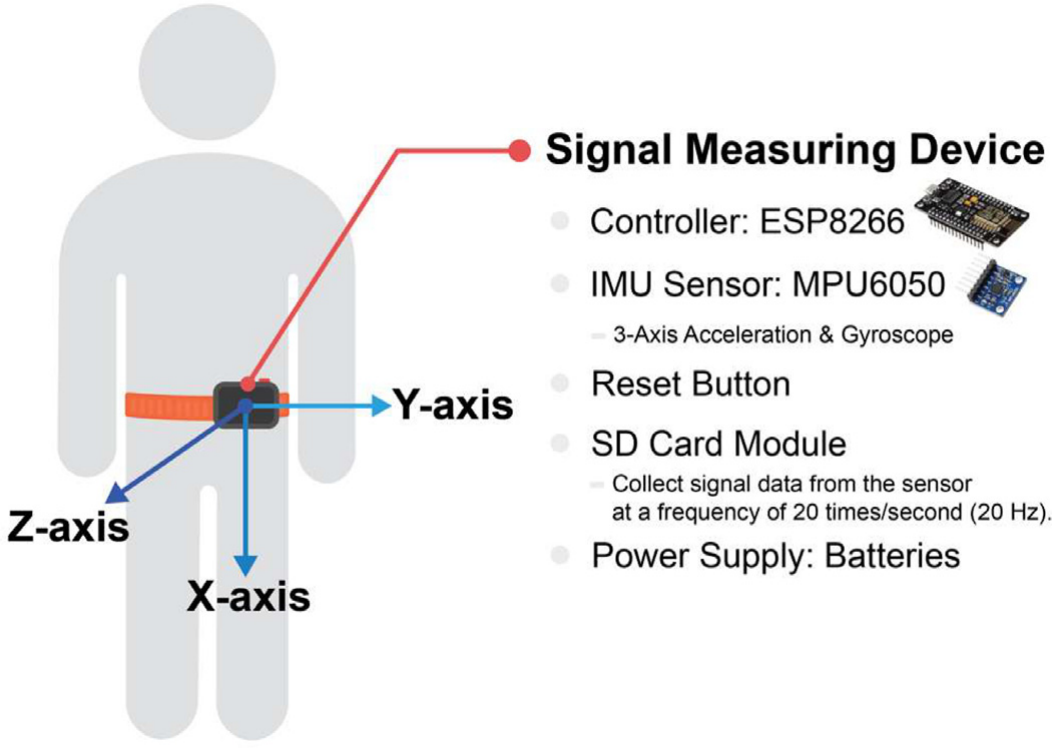
The position of the device on the participant's body.

The position of the device is located at the participant's waist, as shown in Fig. 1. The axes of the acceleration and gyroscope are set as the x-axis being positive in the downward direction, the y-axis being positive toward the left, and the z-axis being positive outward from the participant.

### 4.3 The Montreal Cognitive Assessment (MoCA)

The MoCA is a paper-based screening tool designed to detect early-stage cognitive impairment. This assessment evaluates multiple dimensions of brain function, encompassing intention, attention, executive capabilities, memory, visual construction skills, conceptual thinking, mathematical calculations, and spatial awareness. The scoring system spans from 0 to 30 points. It usually takes 10 to 15 min to complete the test. Having a score of 26 or higher is indicative of normal cognitive function [9].

### 4.4 Clinical Observation Checklist

We have developed this assessment questionnaire to enable experts to evaluate the physical capabilities of participants. The questionnaire categorizes the physical ability tests into four distinct sections according to the TUG protocol. The assessment encompasses the subsequent areas: physical abilities related to sitting-to-standing and standing-to-sitting, with scoring criteria drawn from the Burg Balance Test within the same segment [10]; physical abilities during walking, utilizing a checklist influenced by common gait disorder in the elderly [11]; physical abilities while turning, focusing on time taken and quality of turn such as smoothness or fluidity.

## 5 Data Collection

This experimental protocol was employed to acquire the dataset, comprising 2 stations.

**Fig. 2:**
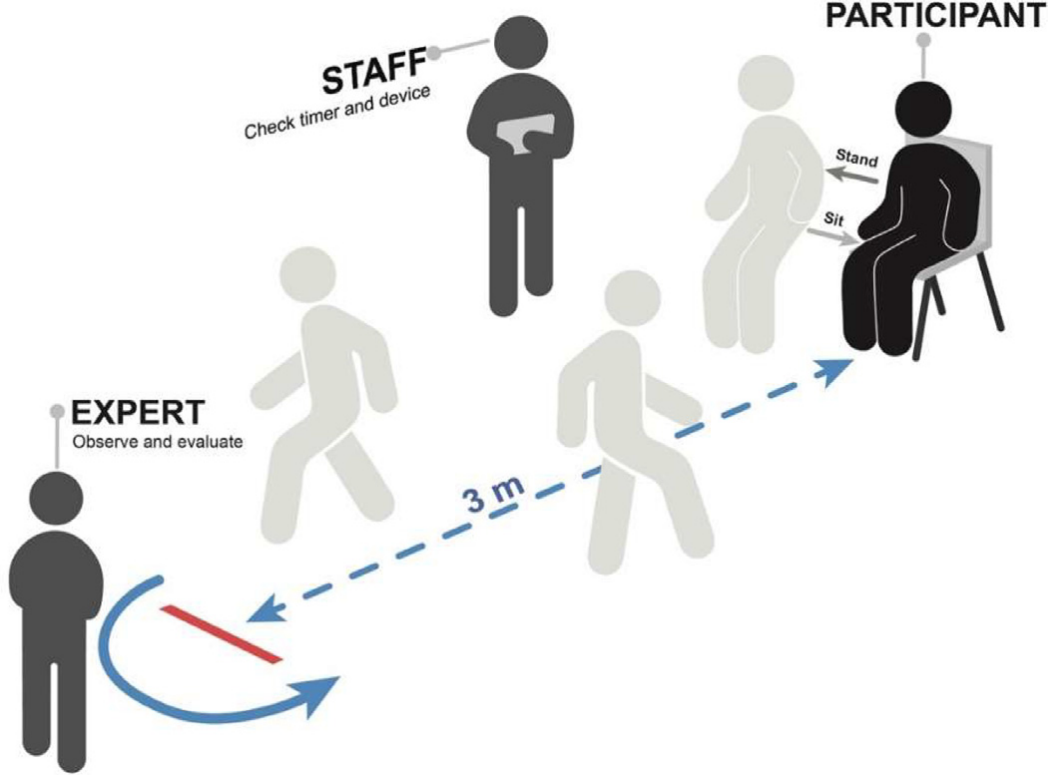
The visualization of the TUG procedure and the location of staff and expert in the second station.

• The first station involved clinical assessment, where an occupational therapist gathered participants' basic information such as age, underlying health conditions, and history of falls. Subsequently, the therapist conducted the MoCA assessment.
• The second station, dedicated to the TUG test, we attach the sensor devices to participants' waists and provide an explanation of the TUG procedure. Participants were required to complete three rounds of the test. Throughout the TUG assessment, three specialists closely observed participants' physical performance, utilizing a clinical observation form. The visualization of the second station is shown in Fig. 2.

## 6 Data Processing

The raw signal data collected during participants performing TUG were recorded from the period that the staff switched on the device on the participant's waist until the participants sat down. We pre-processed the data by removing the signal during the preparatory stage of the task and after the end of the trial. The method used for processing the signal is finding the combined magnitude of the signal from acceleration and gyroscope to determine the start point of signal data through the equation of Acceleration Magnitude = (a_x^2 + a*_*y^2 + a*_*z^2) ^0.5. The threshold was set as any time step with a difference greater than 0.04 at the start of walking, and then we used this threshold to determine the start of the trial. The end time step is estimated using the gyroscope x-axis signal data instead of the acceleration magnitude data, as it has less noise in the visualization. The equation used to determine the end point of walking is: End = Start + (Duration/Sampling Rate), with the sampling rate being 0.05 s for sensor data.

**Fig. 3:**
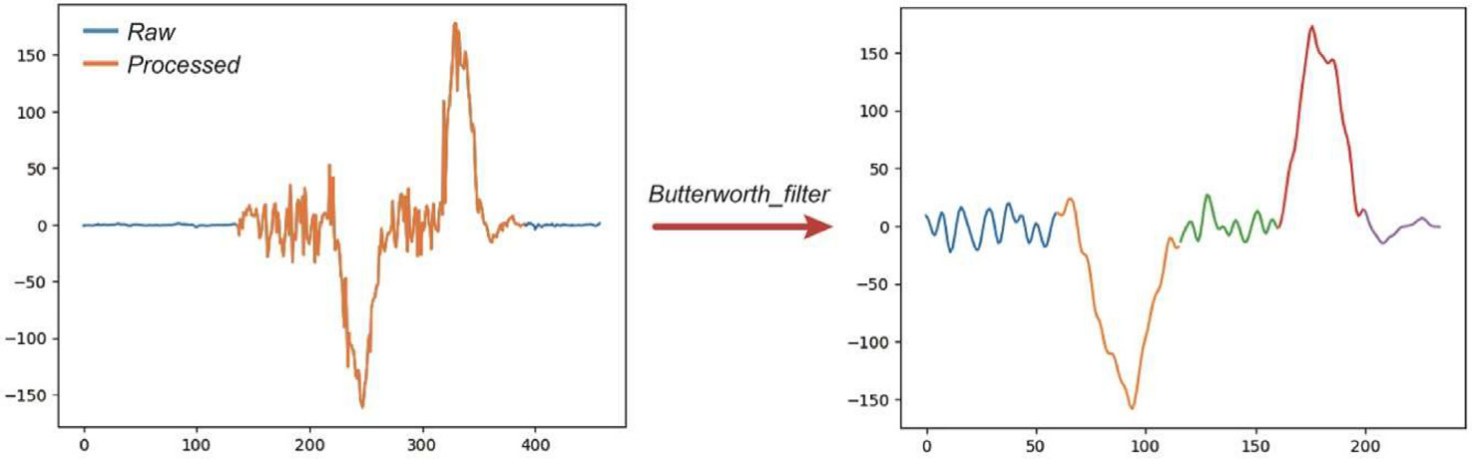
Illustration of the pre-processing gyroscope signal data.

To reduce the noise in the signal data, we applied the Butterworth filter, which is a type of signal processing filter designed to have as flat frequency response as possible in the pass band. Consequently, we obtained the final processed data that we are providing in the dataset. An illustration of the data processing outcome is shown in Fig. 3.

**Fig. 4:**
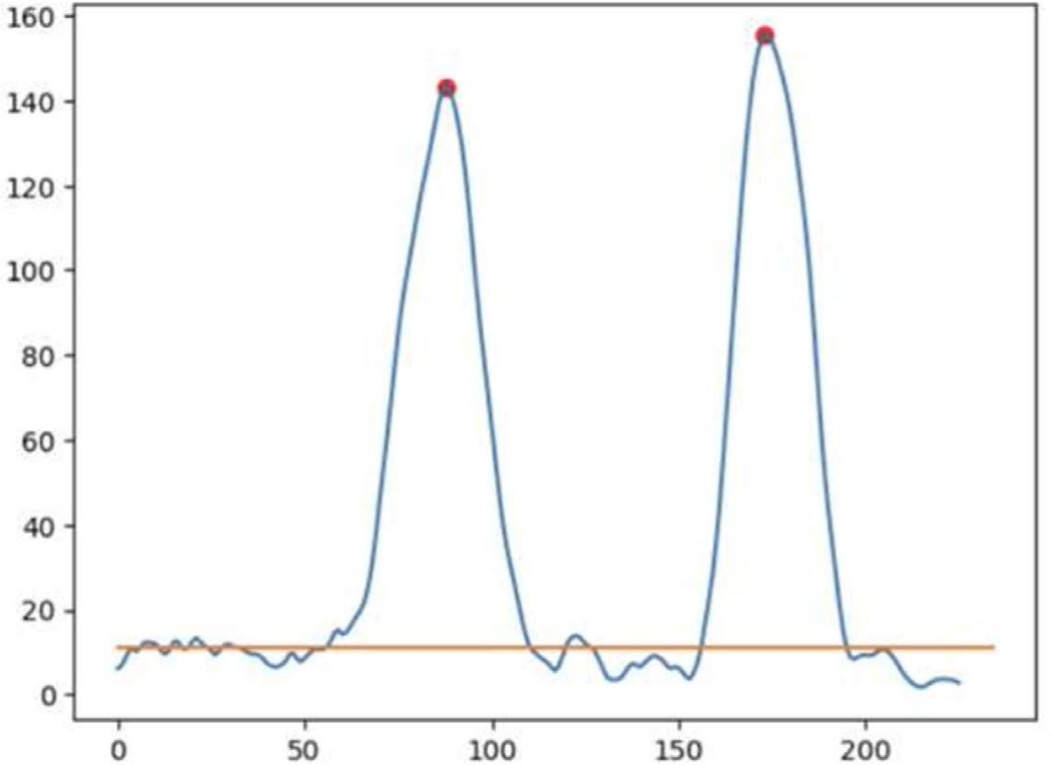
An illustration of two maxima of moving averages, with a threshold set at the second quartile value.

**Fig. 5:**
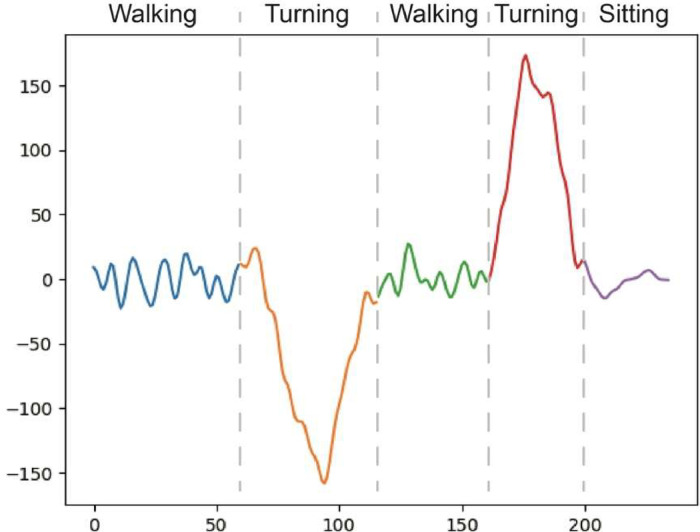
Sample segmented g_x signal data.

After the signal data passed the low-pass filter, we segmented the g_x signal, giving clear visual separations between walking and turning. The segmentation step starts with finding the moving averages of the absolute values of the filtered g_x data with a window width of 10 time steps. Computing the moving averages created a smoothened time-series data with clear local maxima that denote turning events. Then, the two highest local maxima on the moving average graph would be individually located as the two separate turning periods. Starting from each of these local maxima, we denoted every time step to the left and the right of the local maxima to be "turn" data. If the smoothened g_x value is less than the second quartile value (or 50 percentile), it is considered "non-turn" data, as shown in Fig. 4. Then, we segmented and visualized the signal into three phases, as shown in Fig. 5. The detail of each phase is as follow:

• **Walking Phase**: This phase is when participants walk from the start point to the turning point indicated as the blue signal (walk1) and walk from the turning point back to the start point indicated as the green signal (walk2). Segmentation was done on the signal data before the first “non-turn”/“turn” and Signal data before the first "non-turn"/"turn.”
• **Turning Phase**: When participants turn at a turning point, it can be a rightward turn or a leftward turn, depending on the individual preference. The first turn is at the turning point three meters away from the chair (or the red line in Fig. 2), and it is indicated as the orange curve (turn1). The second turn is at the starting point (turn to sit down), indicated as the red line (turn2). Segmentation was done on the signal data between the first and the second “non-turn”/“turn” and between the third and fourth “non-turn”/”turn”.
• **Sitting Phase**: This phase is defined after the participants completely turn around at the starting point and then sit down. It is indicated as the purple curve (sit), and it is segmented from the signal data after the fourth “non-turn”/”turn.”

## Limitations

No timestamp was applied during the acquisition of signal data. Certain participants exhibited movements extending beyond the typical sitting duration or adopted a gradual sitting pace, leading to elongated signal patterns toward the end of their recordings. Additionally, participants displayed variability in the direction of their turns, with some favoring rightward turns while others opted for leftward turns. This discrepancy in turning preferences is attributed to individual variations in rotational tendencies, consequently influencing distinct signal characteristics. Hence, discernible dissimilarities manifest between the signal patterns associated with left and right turns.

## Ethics Statement

The research adhered to the ethical guidelines established for medical investigations involving human participants in accordance with the Declaration of Helsinki by the World Medical Association. The ethical aspects of the research underwent a comprehensive evaluation and received the official endorsement of the Ethics Committee at King Mongkut's University of Technology Thonburi, reference number KMUTT-IRB-COA-2022–008. All participants granted their explicit consent in writing prior to their involvement in the study.

## CRediT authorship contribution statement

**Wisanu Jutharee:** Conceptualization, Methodology, Investigation, Resources. **Chatchai Paengkumhag:** Methodology, Investigation, Writing – original draft. **Warissara Limpornchitwilai:** Methodology, Investigation, Formal analysis, Visualization, Writing – original draft. **Wen Tao Mo:** Formal analysis, Data curation, Validation. **Jonathan H. Chan:** Supervision, Writing – review & editing. **Tanagorn Jennawasin:** Methodology, Investigation, Software, Formal analysis. **Boonserm Kaewkamnerdpong:** Conceptualization, Resources, Project administration, Writing – review & editing.

## Data Availability

Fall Risk Assessment Dataset: Older-Adult Participants undergoing the Time Up and Go Test (Original data) (Mendeley Data).

## References

[1] “UN decade of healthy ageing: plan of action 2021-2023,” United Nations.

[2] Renfro M., Maring J., Bainbridge D., Blair M. (2016). Fall risk among older adult high-risk populations: a review of current screening and assessment tools. Curr. Geriatr. Rep..

[3] Soomar S.M., Dhalla Z. (2023). Injuries and outcomes resulting due to falls in elderly patients presenting to the Emergency Department of a tertiary care hospital – a cohort study. BMC Emerg. Med..

[4] Huang W.-Y., Huang H., Wu C.-E. (2022). Physical activity and social support to promote a health-promoting lifestyle in older adults: an intervention study. Int. J. Environ. Res. Publ. Health.

[5] Barmentloo L.M., Dontje M.L., Koopman M.Y., Olij B.F., Oudshoorn C., Mackenbach J.P., Polinder S., Erasmus V. (2020). Barriers and facilitators for screening older adults on fall risk in a hospital setting: perspectives from patients and healthcare professionals. Int. J. Environ. Res. Publ. Health.

[6] Choi A., Kim T.H., Yuhai O., Jeong S., Kim K., Kim H., Mun J.H. (2022). Deep learning-based near-fall detection algorithm for fall risk monitoring system using a single inertial measurement unit. IEEE Tran. Neural Syst. Rehabil. Eng..

[7] Lwanga S., Lemeshow S. (1991). Sample size determination in health studies: a practical manual. World Health Organ..

[8] Zakaria N.A., Kuwae Y., Tamura T., Minato K., Kanaya S. (2015). Quantitative Analysis of Fall Risk Using TUG Test. Comput. Methods Biomech. Biomed. Eng..

[9] Mocatest, “Moca,” March 2007. [Online]. Available: www.mocatest.org. [Accessed 15 September 2023].

[10] Muir S.W., Berg K., Chesworth B., Speechley M. (2008). Use of the berg balance scale for predicting multiple falls in community-dwelling elderly people: a prospective study. Phys. Ther..

[11] Pirker W., Katzenschlager R. (2017). Gait disorders in adults and the elderly: a clinical guide. Wiener klinische Wochenschrift.

